# CurSa: scripts to curate metadata and sample genomes from GISAID for analysis and display in nextstrain and microreact

**DOI:** 10.1093/biomethods/bpad007

**Published:** 2023-04-17

**Authors:** Luis Delaye

**Affiliations:** Department of Genetic Engineering, CINVESTAV-Irapuato, Irapuato, Guanajuato 36824, Mexico

**Keywords:** coronavirus, SARS-CoV-2, phylogenomics

## Abstract

The coronavirus SARS-CoV-2 is the most sequenced pathogen ever, with several million genome copies deposited in the GISAID database. This large amount of genomic information poses non-trivial bioinformatic challenges for those interested in studying the evolution of SARS-CoV-2. One common problem when studying the phylogeny of the coronavirus in its geographical context is to count with accurate information of the location of the samples. However, this information is filled by hand by research groups all over the world and sometimes typos and inconsistencies are introduced in the metadata when submitting the sequences to GISAID. Correcting these errors is laborious and time-consuming. Here, we provide a suite of Perl scripts designated to facilitate the curation of this vital information and perform a random sampling of genome sequences if necessary. The scripts provided here can be used to curate geographic information in the metadata and sample the sequences from any country of interest to ease the preparation of files for Nextstrain and Microreact, thus accelerating evolutionary studies of this important pathogen. CurSa scripts are accessible via: https://github.com/luisdelaye/CurSa/.

## Introduction

Current sequencing technologies allowed the scientific community to describe the evolution of SARS-CoV-2 with unprecedented detail [1]. At the time of writing, there are more than 15 million SARS-CoV-2 genome sequences deposited in GISAID (https://www.gisaid.org). This large amount of genomic information is at the same time a milestone and a millstone for those interested in studying the evolution of SARS-CoV-2.

The scientific community all over the world has developed powerful bioinformatic tools to facilitate the evolutionary analysis of large quantities of genome sequences [2]. Nextstrain and Microreact are two popular and fine platforms used by the community to study and visualize the evolution of pathogens in a geographical context [3, 4]. The two main components of Nextstrain are Augur and Auspice. The first one is a pipeline that connects several tools via Snakemake to analyze genomic data and the second is used to visualize the results. Microreact is a platform to visualize the evolution of pathogens through time in a geographical context.

One of the challenges that are faced when attempting a phylogeographic study of SARS-CoV-2 is to count with accurate information regarding the geographic localization of the samples. This information is found in the metadata associated with genome sequences in the GISAID database and is fulfilled by research groups all over the world. This process is not error proof and sometimes typos are introduced as well as inconsistencies. For instance, the name of the same city can be written in more than one way; like ‘Mexico City’ which is sometimes written as ‘CDMX’ and in other occasions as ‘Ciudad de Mexico’ or ‘Mexico DF’ (not to mention typos like ‘CMX’).

These inconsistencies impair the performance of Nextstrain because geographic localities showing varying names in metadata and reference files from Augur will not be properly georeferenced. A similar situation may apply to Microreact. Correcting these errors is laborious and time-consuming, however crucial for proper phylogenetic analysis in a geographical context. Here, we provide a suite of Perl scripts designated to facilitate the curation of this vital information.

Another problem faced by researchers is how to sample an appropriate subset of genome sequences to study the phylogeny of SARS-CoV-2 in a focal country? This is particularly important for countries where large amounts of genomes have been sequenced. Auspice can properly display ∼5000 sequences, however up to November 2022, there are 55 countries with more than 10 000 genome sequences available and 26 of them have more than 50 000 sequences (https://www.gisaid.org). And this number has grown ever since.

Moreover, running a Nextstrain analysis with 4500 sequences on a personal computer (MacPro 3.5 GHz 6-Core Intel Xeon E5, 16 GB 1866 MHz DDR3) takes about 28 h to complete. Attempting a comprehensive Nextstrain analysis in a personal computer is impractical if not impossible for those countries with a large number of sequences. Therefore, scientists attempting an evolutionary study of SARS-CoV-2 face the problem of how to adequately sample a set of input sequences for practical Nextstrain analysis.

Nextstrain, via Augur and accompanying Python scripts, offers strategies to (i) filter and then (ii) sample genome sequences at different geographic resolutions. These are fine strategies that work well and are very practical for a moderate number of sequences. Here, we also provide a script designated to facilitate the sampling of SARS-CoV-2 genome sequences downloaded from GISAID to make a phylogenomic analysis in Nextstrain and their display in Microreact. Our sampling strategy can be used instead of or in combination with the strategies provided by Augur.

## Materials and methods

To overcome the above problems, we developed CurSa which consists of a suite of Perl scripts that are used sequentially: first, to curate the inconsistencies between the names of the geographic localities in the metadata.tsv file downloaded from GISAID and the reference files color_ordering.tsv and lat_longs.tsv from Augur and second, to sample genome sequences for phylogenetic analysis in Augur and their display in Auspice and Microreact. In Table 1, we show the scripts that comprise CurSa and a brief description of their function.

**Table 1:** The suite of scripts comprising CurSa

Script	Function
concatenate_tsv_files.pl	Concatenate all files downloaded from GISAID $ perl concatenate_tsv_files.pl outfiles: outfile.tsv, outfile.fasta
compare_names.pl	Find inconsistencies between metadata.tsv and color_ordering.tsv files $ perl compare_names.pl color_ordering.tsv metadata.tsv Mexico outfile: substitute_proposal.tsv
substitute_names.pl	Creates a new metadata.tsv file by using the information in substitute_proposal.tsv to correct the inconsistencies $ perl substitute_names.pl metadata.tsv substitute_proposal_round1.tsv outfile: outfile.tsv
check_coordinates.pl	Checks whether all localities in color_ordering.tsv have an associated coordinate in lat_longs.tsv $ perl check_coordinates.pl metadata.tsv color_ordering.tsv lat_longs.tsv Mexico outfile: names_lacking_coordinates.txt
format.pl	Formats metadata and sequence files for Augur analysis and Auspice display $ perl format.pl substituted.metadata.tsv sequences.fasta outfiles: formated_metadata.tsv, formated_sequences.fasta
sample.pl	Sample genome sequences for Augur analysis and Auspice display $ perl sample.pl formated_metadata.tsv formated_sequences.fasta 2718 10 2020-01-01 2023-01-01 outfiles: sampled_metadata.tsv, sampled_sequencies.fasta, sampled_report.txt
create_microreact.pl	Create files for Microreact display $ perl create_microreact.pl lat_longs.e1.tsv aligned.fasta metadata.tsv Mexico outfile: outfile_for_microreact.tsv

Next, we describe the rationale behind each one of the scripts that conform CurSa.

concatenate_tsv_files.pl

GISAID allows users to download a maximum of 5000 sequences (and their associated metadata) at once. Depending on the focal country, the number of sequences can be much larger. If the number of sequences from the focal country is larger than 5000, the sequences have to be downloaded in several different batches. This script concatenates all the sequence and metadata files into a single metadata file named outfile.tsv and a single sequence file named outfile.fasta. It also checks that all fasta headers are represented in the metadata.

compare_names.pl and substitute_names.pl

**Figure 1:**
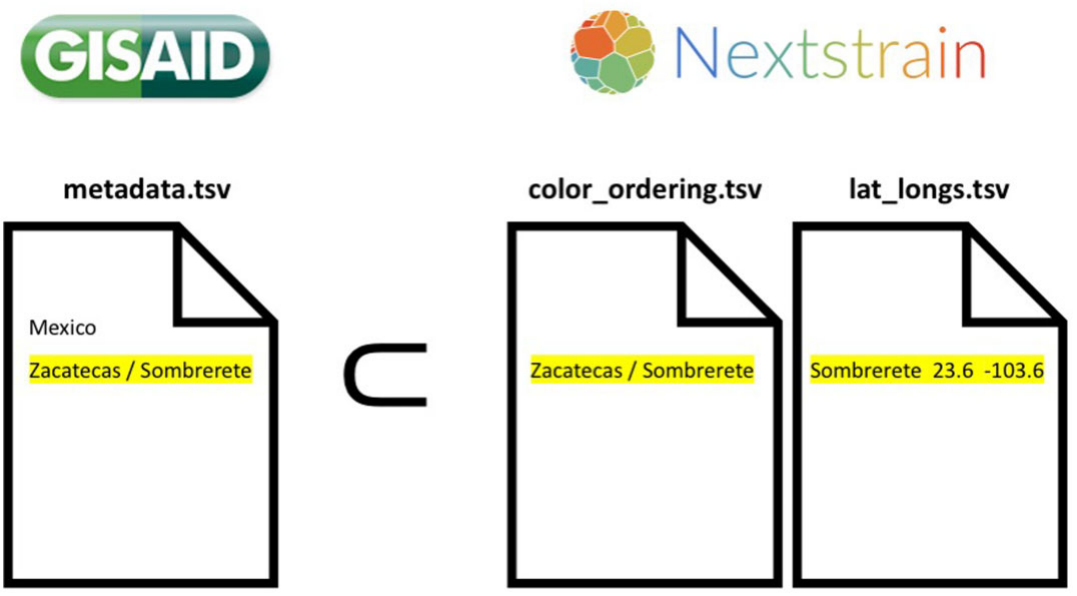
All geographic localities in metadata.tsv from the focal country (in this case, Mexico) have to be found in the reference files color_ordering.tsv and lat_long.tsv. As an example, the city of Sombrerete in the state of Zacatecas is shown.

These scripts are used to curate the names from the geographic localities. Basically, all the geographic localities in the metadata file downloaded from GISAID (metadata.tsv) have to be contained in the reference files color_ordering.tsv and lat_longs.tsv from Augur (Fig. 1).

**Figure 2:**
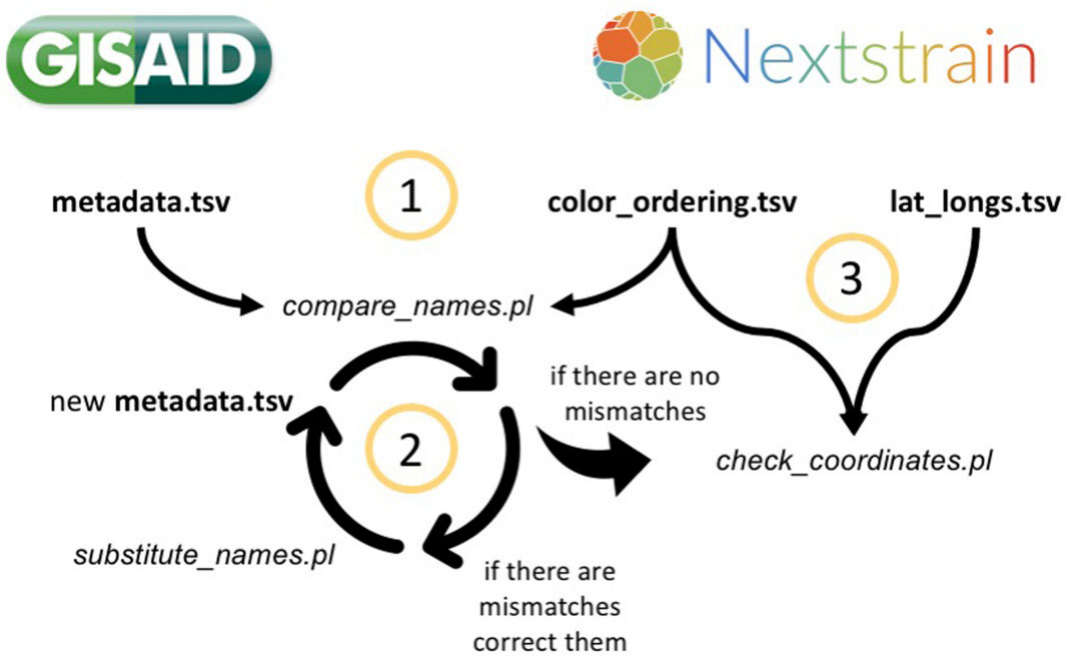
The scripts compare_names.pl and substitute_names.pl are used cyclically until there are no more inconsistencies between metadata.tsv and color_ordering.tsv files. Then, once there are no more inconsistencies, another script (check_coordinates.pl) is used to check if all names from the geographic localities found in color_ordering.tsv have an associate coordinate in lat_longs.tsv.

The first script (compare_names.pl) checks whether the geographic localities in metadata.tsv are contained within color_ordering.tsv. This script outputs a file named substitute_proposal.tsv with the detected inconsistencies. The user then corrects the inconsistencies in this file and then runs the second script (substitute_names.pl) to create a new metadata file without the inconsistencies. The second script uses the information in substitute_proposal.tsv to create the new corrected metadata file (Fig. 2). These scripts can be used cyclically until there are no more inconsistencies.

check_coordinates.pl

This script checks whether all geographic localities within color_ordering.tsv are also found in lat_longs.tsv file. This is done only for the focal country.

format.pl

This script deletes the prefix hCoV-19/ from the field strain (in the metadata) and in the header of the FASTA sequence files. It also checks whether there are no duplicate records with the same strain. If there are, it keeps the most recent one. This script is similar to sanitize_metadata.py that comes with Augur.

sample.pl

**Figure 3:**
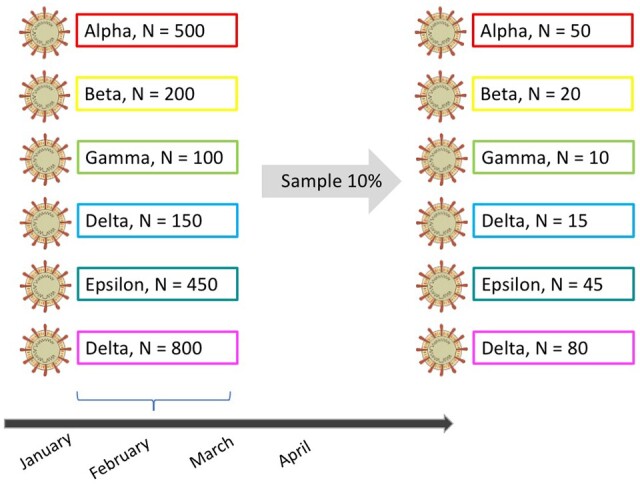
The script sample.pl samples a percentage of genomes (specified by the user) on a monthly basis. The script requires a data range and a seed to generate random numbers to sample the genomes. The sample is taken on a monthly basis irrespective of the coronavirus variant.

Nextstrain provides via Augur a method to filter sequences from specific regions and/or dates. Here, we provide a simple alternative to the method provided by Augur to subsample sequences for Nextstrain analysis. The script sample.pl requires a percentage of genomes to sample on a monthly basis, and the date ranges (Fig. 3). The script also requires a seed to generate random numbers to sample the sequences. This allows the user to have control over the sampling process (i.e. if the same seed is provided, the same set of genomes will be sampled each time the script is executed on the same files, facilitating repeatability).

create_microreact.pl

Once the genome sequences have been analyzed with Nextstrain (via Augur) and a multiple sequence alignment and a phylogenetic tree are generated, the script create_microreact.pl can be used to generate the file that is required by Microreact to display the sequences in a geographical context.

**Figure 4:**
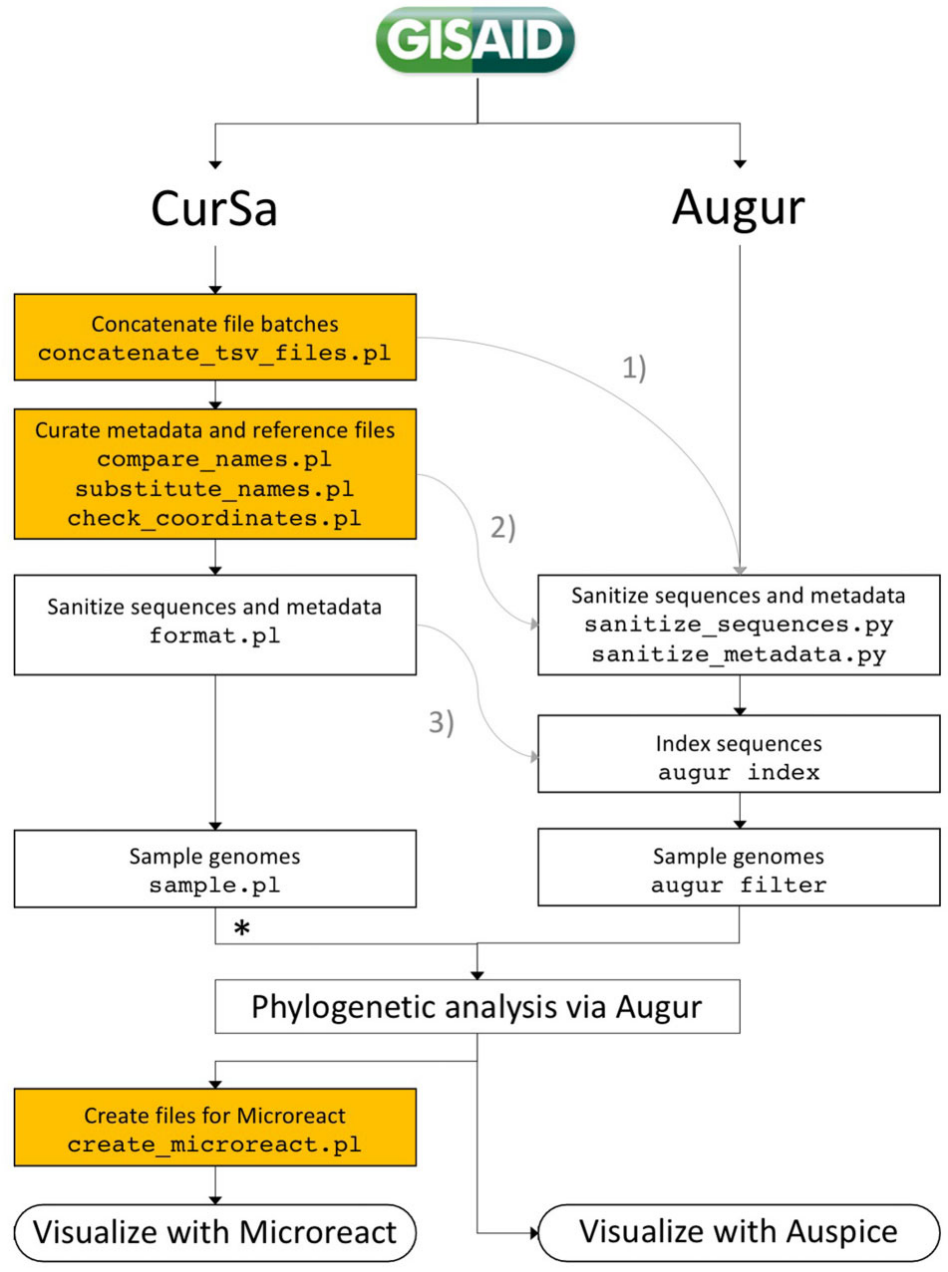
Roadmap of CurSa scripts and how they can be used in combination with Augur. Functionalities provided by CurSa not present in Augur are shown in orange. Gray arrows show the three optional paths that connect CurSa with Augur pipelines. *Sequences will have to be indexed prior phylogenetic analysis.

CurSa scripts can be used in combination with those provided by Nextstrain/Augur (Fig. 4). For instance, if sequence files and metadata are downloaded in several different batches from GISAID, it is possible to use the script concatenate_tsv_files.pl to concatenate them and then proceed to curate the metadata and reference files via CurSa or move directly to the Augur pipeline (number 1 in Fig. 4). It is also possible to curate the metadata and reference files via CurSa and then proceed to sanitize the metadata and sequence files in the Augur pipeline (number 2 in Fig. 4). Another possibility is to sanitize the metadata and sequence files with format.pl and then index and sample the genomes with Augur (number 3 in Fig. 4). Finally, it is possible to go all the way through CurSa and then perform the phylogenetic analysis in Augur. This flexibility allows to combine the functionalities provided by CurSa with those of Augur.

## Results

Next, we show an example of how we used CurSa scripts to create Mexstrain (https://ira.cinvestav.mx/mexstrain/). We provide a detailed description of each one of the steps in Supplementary File S1.

### Download data

On 3 February 2023, we downloaded all complete, high coverage and ‘collection date complete’ sequences sampled in Mexico from GISAID database (https://gisaid.org). The sequences and their associated metadata were downloaded in the format required by Augur (i.e. input for the Augur pipeline). Because GISAID allows to download a maximum of 5000 entries at once, and there were 35 633 sequences from Mexico, we downloaded the information from each Mexican state separately and then used the script concatenate_tsv_files.pl to concatenate all the information into a single metadata and sequence files (Supplementary File S1, Result S1).

### Curate metadata and reference files

Next, we proceeded to curate the metadata.tsv and the reference files color_ordering.tsv and lat_longs.tsv. As a first step, we manually check the color_ordering.tsv file to identify simple typos in the names of localities from Mexico. This is important because the names in color_ordering.tsv will be used as gold-standards when compared with those in metadata.tsv and lat_longs.tsv files.

Once the names in color_ordering.tsv were manually checked, we proceeded to run compare_names.pl to identify inconsistencies between metadata.tsv and color_ordering.tsv (Supplementary File S1, Result S2). By this we identified 27 localities in metadata.tsv without a correspondence in color_ordering.tsv. These 27 inconsistencies mapped to 2445 entries/genomes in the metadata.tsv file (∼7% of all sequences from Mexico). Most of the inconsistencies were from ‘Estado de Mexico’ that in metadata.tsv is written as ‘State of Mexico’ (2188 out of 2290 times).

The script compare_names.pl outputs a file named substitute_proposal.tsv. This file contains all detected inconsistencies. We renamed this outfile to substitute_proposal_round1.tsv and manually modified it by using a simple text editor to correct the inconsistencies (see Supplementary File S1).

Once all inconsistencies have been corrected in substitute_proposal_round1.tsv, we used the script substitute_names.pl to create a new metadata file without detected errors. This script reads the content of substitute_proposal_round1.tsv and uses it to create the new metadata file named outfile.tsv. This new metadata file is temporarily renamed as metadata_round1.tsv.

A second run of *compare_names.pl* revealed that there are no more inconsistencies in the new metadata file metadata_round1.tsv. We renamed the metadata_round1.tsv to substituted.metadata.tsv.

Finally, we used the script check_coordinates.pl to check if all geographic localities in color_ordering.tsv have a coordinate in lat_logns.tsv. By this, we identified 16 localities from Mexico in color_ordering.tsv (that were also in substituted.metadata.tsv) without coordinates in lat_logns.tsv file. We manually added the lacking coordinates to lat_logns.tsv file.

### Formatting metadata and genome sequences

The script format.pl strips the prefix “hCov-19/” from the strain id in the substituted.metadata.tsv file and from the headers of the sequence FASTA file (Supplementary File S1, Result S3). The script also resolves duplicate entries sharing the same strain id in the metadata file by keeping the most recent one.

### Sampling genomes

As described above, the script sample.pl samples a custom percentage of genomes on a monthly basis. For instance, we asked for a 10% sample of genomes from each month since the first record in Mexico (Supplementary File S1, Result S4). By this, we sampled 3564 genomes out of 35 633. This script outputs two files sampled_metadata.tsv and sampled_sequences.fasta.

### Nextstrain analysis via Augur and display with Auspice

**Figure 5:**
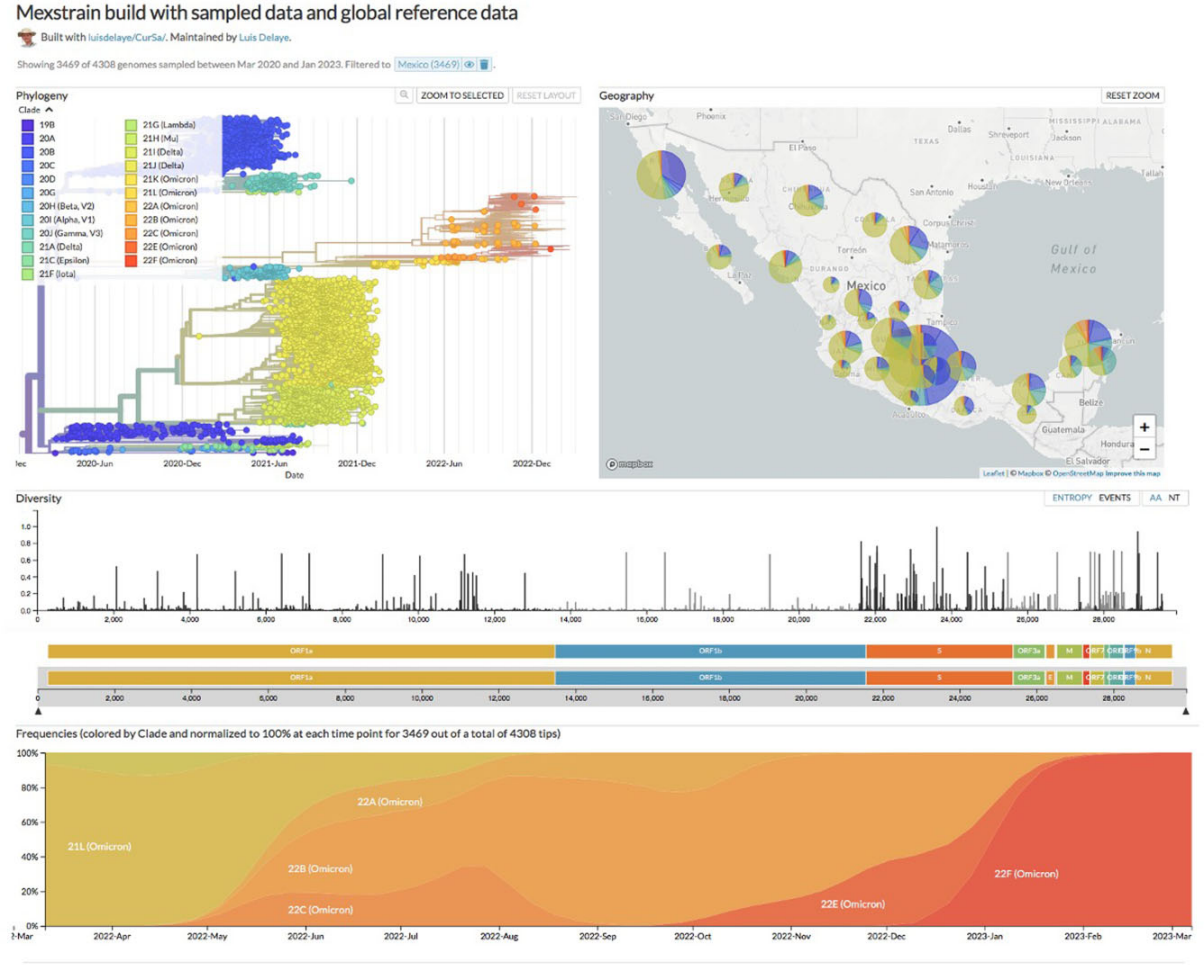
Phylogenetic analysis of sampled sequences inferred with Nextstrain. Sequences from Mexico are indicated with circles.

Finally, we copied the outfiles obtained with sample.pl to the data/ directory and the curated files color_ordering.tsv and lat_longs.tsv to the defaults/ directory within the local Nextstrain build. And we configured Auspice to contextualize the genome sequences from Mexico with those GenBank sequences of the Global sample provided by Nextstrain (Supplementary File S1, Result S5). The result is shown in Fig. 5.

### Visualize analysis in Microreact

**Figure 6:**
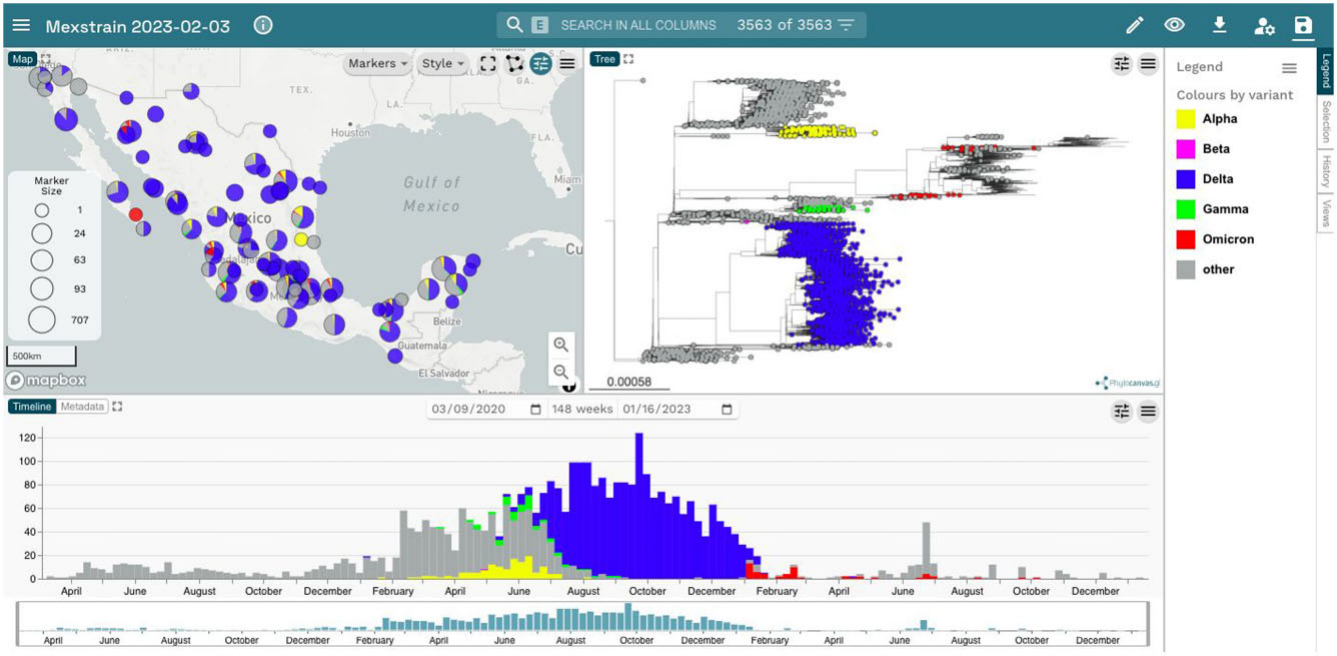
Sampled sequences can be visualized in Microreact all together with the phylogeny inferred with Nextstrain.

To display the phylogenetic analysis performed with Nextstrain in Microreact, we executed the script create_microreact.pl (Supplementary File S1, Result S6). This script runs on the files: lat_longs.tsv, aligned.fasta, and sampled_metadata.tsv. The lat_longs.tsv and sampled_metadata.tsv files were described previously. The aligned.fasta file is the result of running Nextstrain on the sampled set of sequences and is found in ncov/results/yourconfigfile/ within the local Nextstrain build. This script will create the files: outfile_for_microreact.tsv. This file contains the table required by Microreact with all the sequences found in metadata.sampled.tsv. The tree file tree_raw.nwk required by Microreact is found in ncov/results/yourconfigfile/. We uploaded the sampled_metadata.tsv and the tree_raw.nwk to Microreact (https://microreact.org) to visualize the data with its phylogeny (Fig. 6).

### Applications

Phylogenetic analyses in Nextstrain assisted by CurSa scripts were used to describe the diversity of SARS-CoV-2 prior vaccination stages in Mexico [5]; and are used to maintain Mexstrain (https://ira.cinvestav.mx/mexstrain/) a web page designated to facilitate the visualization of the phylogeny of SARS-CoV-2 in Mexico. Here, we make CurSa available to the scientific community by carefully describing its functionality and how it can be used to extend and complement those of Augur to improve the accuracy of phylogenomic analysis in a geographical context.

## Conclusions

CurSa is a suite of Perl scripts designated to curate metadata files and sample genome sequences from SARS-CoV-2 (that were downloaded from GISAID) for phylogenomic analysis in Nextstrain and display in Microreact. CurSa scripts allow the user to sample a moderate number of sequences to run Nextstrain on a personal computer and to correct inconsistencies in metadata files regarding the geographic location of the samples. As far as we know, there is no other software designed to facilitate the curation of geographic localities in metadata from SARS-CoV-2. The set of sequences sampled with CurSa are a starting point to more sophisticated subsampling methods implemented in Nextstrain if desired. All CurSa scripts are properly commented and should be crystal clear to any Perl programmer. Overall, CurSa is aimed to facilitate and accelerate evolutionary studies in SARS-CoV-2.

## Supplementary Material

bpad007_Supplementary_Data

## Data Availability

CurSa scripts are accessible via Github at: https://github.com/luisdelaye/CurSa/. Detailed instructions on how to use CurSa scripts are provided via GitHub.
